# Household costs of dengue illness: secondary outcomes from a randomised controlled trial of dengue prevention in Guerrero state, Mexico

**DOI:** 10.1186/s12889-017-4304-x

**Published:** 2017-05-30

**Authors:** José Legorreta-Soberanis, Sergio Paredes-Solís, Arcadio Morales-Pérez, Elizabeth Nava-Aguilera, Felipe René Serrano-de los Santos, Diana Lisseth Dimas-Garcia, Robert J Ledogar, Anne Cockcroft, Neil Andersson

**Affiliations:** 1 Centro de Investigación de Enfermedades Tropicales de la Universidad Autónoma de Guerrero, Acapulco, Guerrero Mexico; 2 CIETinternational, New York, NY USA; 3 Department of Family Medicine, McGill University, Montreal, Canada; 4 CIET Trust, Gaborone, Botswana

**Keywords:** Dengue, Costs, Randomised controlled trial

## Abstract

**Background:**

Dengue is a serious public health problem with an important economic impact. This study used data from a cluster randomised controlled trial of community mobilisation for dengue prevention to estimate the household costs of treatment of dengue illness. It examined the economic impact of the trial intervention in the three coastal regions of Mexico’s Guerrero State.

**Methods:**

The 2010 baseline survey covered households in a random sample of 90 clusters in the coastal regions; the clusters were randomly allocated to intervention or control and re-surveyed in 2012. The surveys asked about dengue cases in the last 12 months, expenditures on their treatment, and work or school days lost by patients and care givers. We did not assign monetary value to days lost, since a lost day to a person of low earning power is of equal or higher value to that person than to one who earns more.

**Results:**

The 12,312 households in 2010 reported 1020 dengue cases in the last 12 months (1.9% of the sample population). Most (78%) were ambulatory cases, with a mean cost of USD 51 and 10.8 work/school days, rising to USD 96 and 11.4 work/school days if treated by a private physician. Hospitalised cases cost USD 28–94 in government institutions and USD 392 in private hospitals (excluding additional inpatient charges), as well as 9.6–17.3 work/school days. Dengue cases cost households an estimated 412,825 work/school days throughout the three coastal regions. In the follow up survey, 6.1% (326/5349) of households in intervention clusters and 7.9% (405/5139) in control clusters reported at least one dengue case. The mean of days lost per case was similar in intervention and control clusters, but the number of days lost from dengue and all elements of costs for dengue cases per 1000 population were lower in intervention clusters. If the total population of the three coastal regions had received the intervention, some 149,401 work or school days lost per year could have been prevented.

**Conclusion:**

The economic effect of dengue on households, including lost work days, is substantial. The Camino Verde trial intervention reduced household costs for treatment of dengue cases.

**Trial registration:**

The trial was registered as ISRCTN:27,581,154.

**Electronic supplementary material:**

The online version of this article (doi:10.1186/s12889-017-4304-x) contains supplementary material, which is available to authorized users.

## Background

Since its re-emergence in the Americas, dengue continues to proliferate in all its clinical manifestations despite prevention efforts [1]. Treatment of dengue illness consumes considerable resources in Latin America [2]. There is a need to study further the economic burden that dengue imposes on households [3]. In Mexico, the Specific Action Program for Dengue 2013–2018 (Programa de Acción Específico. Prevención y Control de Dengue 2013–2018) confirmed a protocol to identify dengue fever patients as potential carriers of the disease, and to register them in the National Epidemiological Surveillance System (Sistema Nacional de Vigilancia Epidemiológica) to locate cases in time and space [4].

A number of studies have estimated the economic impact of dengue illness, including direct costs of ambulatory and hospital cases and indirect costs of the loss of productive time caused by the illness [5–9]. These studies report costs from a societal perspective which takes into account household costs and costs to the government through free or subsidized health services. This perspective also assigns a monetary value to work or school time lost by dengue sufferers and their caregivers, a value that varies according to their potential earning power.

In this article we report an analysis of the costs of dengue illness in Mexico from a community learning perspective, using data collected from households during the Camino Verde trial: a cluster randomised controlled trial of community mobilisation for dengue prevention in Mexico and Nicaragua [10]. The Camino Verde trial intervention was based on sharing evidence about dengue and its costs with households and communities to motivate collective action against the disease. From this perspective, monetarizing the value of days or school time lost is less relevant. A day of work or school lost to a person of low earning power is of equal or even higher value to that person than it is to one who earns more. We therefore report work or school time lost by dengue patients and their caregivers in simple numbers of days.

This article has two specific objectives. First, we examine the direct and indirect costs to households in a dengue-endemic area, using data from the baseline survey of the Mexican arm of the Camino Verde trial, which took place in 90 clusters representative of the population in the coastal regions of Guerrero State [10]. The second objective is to estimate the impact of the Camino Verde intervention in Mexico in terms of reduced financial burden on the households in the trial intervention clusters compared with control clusters. The evidence we offer about the high direct and indirect costs of dengue illness, and the reduction of such costs by the interventions of the trial, can provide additional incentives for individuals and communities to participate in household and community efforts to reduce dengue illness.

## Methods

The methods of the Camino Verde trial are described in detail elsewhere [10]. In Mexico, the baseline survey in 2010 covered a random sample of 90 representative clusters from the three coastal regions of Guerrero State. The clusters were then randomly allocated to intervention or control arms and re-surveyed in 2012. Dengue is endemic throughout the three coastal regions of Guerrero where the trial took place [4]. It is more common in Acapulco than the other two regions, reflecting the high proportion of urban households in this region.

### Estimating the costs of care for dengue illness

The questionnaire for both the baseline and follow-up surveys included questions about dengue illness in household members during the previous 12 months. We identified self-reported cases of dengue illness from responses to a direct question about each household member in turn “Did this person suffer from dengue in the last year?” For each case we recorded: the sex and age of the patient; the facility where the person was treated; whether she or he was hospitalized or not; the cost to the household for the treatment (consultation, prescribed medications and transport); and the number of days of work or school lost as a consequence of the illness by the patient and the caregiver (if any). We did not record lost school and work days separately.

The questions about the cost to the household for treatment did not include the costs of hospitalization for either private or public hospitals, beyond the costs of consultations, prescribed medications and transport. There are a few additional costs in some of the public hospitals, such as special equipment, but there are important additional costs in private hospitals, including investigations, special procedures, and bed-day charges.

### Extrapolation of costs to the population of Guerrero coastal regions

We extrapolated estimates of dengue frequency, costs of treating dengue illness, and days of work/school lost due to the illness and care by family members from the household sample to the entire population in each of Guerrero’s three coastal regions, starting with the 2010 population (Acapulco 789,971, Costa Grande 413,793 and Costa Chica 428,501) [11].

### Statistical analysis

Analysis relied on CIETmap, an open source software package with a Windows-like interface for the open source statistical programing language R [12]. We calculated means and standard deviations for reported costs of dengue treatment and measured significance of differences in costs between sub-groups using the non-parametric Kruskall-Wallis test. We used a cluster t-test to test for significance of differences of proportions of households with dengue cases and costs of treatment between intervention and control clusters in the follow up survey.

## Results

### Household costs of treating dengue illness

We analysed data from 12,312 households in the baseline survey. The total number of dengue cases reported in the last 12 months was 1020, representing 1.9% of the sample population. Among these cases, the household respondent reported 6.8% (69/1013) as dengue haemorrhagic fever. The period prevalence was the same in males (1.9%, 485/26,117) and females (1.9%, 535/28,280). Dengue cases were more common in Acapulco region (3.5%; 660/18,997) than in Costa Grande (1.5%; 251/17,063) or Costa Chica (0.6%; 109/18,342).

Most (78%; 752/960) of the reported dengue cases were treated at home or as ambulatory patients; only 22% (208/960) were hospitalized. Rates of hospitalization among the cases were 21.4% (131/612) for Acapulco, 19.5% (47/221) for Costa Grande, and 28% (30/107) for Costa Chica. Ambulatory dengue cases were most commonly treated by the *Secretaría de Salud* (SSA) (41%, 303/739) and by private physicians (30%, 222/739). Smaller proportions were treated by other government health institutions: *Instituto Mexicano del Seguro Social* (IMSS) treated 16% (115/739), *Instituto de Seguridad y Servicios Sociales de los Trabajadores del Estado* (ISSSTE) 3% (24/739), and *Secretaría de Defensa Nacional* (SEDENA) 0.5% (4/739). Some 3% (22/739) were treated at pharmacies and 6% (45/739) were treated only at home.

The hospitalization rates for dengue cases treated by different health institutions were as follows: IMSS 37% (70/188), SSA 15% (52/388), ISSSTE 40% (16/40), SEDENA 71% (10/14), pharmacy 8% (2/25) and private physician 20% (55/281).

Table 1 shows the reported household costs for ambulatory dengue cases, both cash expenditures and work or school days lost. The mean household expenditure for ambulatory dengue cases was USD 51 (*n* = 737, SD USD 85.0), with a mean of 10.7 days lost. The lowest expenditure was for those who were treated only at home (USD12.80) and the highest was for cases treated by private physicians (USD 95.50). Those treated at home lost the fewest work/school days (6.5), while those treated at pharmacies lost the most (14.1 days).

**Table 1:** Household costs for treatment of ambulatory dengue cases reported by 12,312 households in 2009–2010 baseline survey

Where treated	Reported expenditure by household (USD)				Work or school days lost			
	No. cases spending anything	Reported expenditure per case (USD)			No. cases with any days lost	Reported days lost per case		
		Mean	SD	Total		Mean	SD	Total
At home only	45	12.7	25	573	46	6.5	5.2	299
IMSS	115	26.5	34	3055	118	10.4	6.4	1227
SSA	303	32.1	56	9735	306	10.6	7.5	3244
ISSSTE	24	48.0	48	1152	24	13.0	8.9	312
SEDENA	4	28.0	37	112	4	8.7	5.4	35
Pharmacy	24	70.0	112	1680	23	14.1	8.3	324
Private doctor	222	95.0	120	21,186	225	11.4	8.3	2569
Total	737	50.87		37,493	746	10.8		8010

IMSS *Instituto Mexicano del Seguro Social*; SSA *Secretaría de Salud*; ISSSTE *Instituto de Seguridad y Servicios Sociales de los Trabajadores del Estado*; SEDENA *Secretaría de Defensa Nacional*; *SD* Standard deviation

Table 2 shows the reported household expenditures for hospitalized dengue cases. The lowest expenditure was reported by those who were hospitalized by SEDENA (USD 28) and the highest was for those hospitalized in private institutions (USD 392). The average number of work or school days lost by patient and caregiver(s) combined was 15.4 (*n* = 203, SD 8.9). This is higher than the number of days lost by dengue cases that were not hospitalized, who lost an average of 10.7 work or school days (*n* = 746, SD = 7.6) (Table 1). Of the 55 dengue cases treated as inpatients in private hospitals, only three came from households where the household head was reported as not in formal employment.

**Table 2:** Household costs for treatment of hospitalized dengue cases reported by 12,312 households in 2009–2010 baseline survey

	Reported expenditure by household (USD)				Work or school days lost			
Where treated	No. cases spending anything	Reported expenditure per case (USD)			No. cases with any days lost	Reported days lost per case		
		Mean^a^	SD	Total		Mean^b^	SD	Total
IMSS	70	84	132.4	5889	70	14.6	8.0	1022
SSA	50	94	114.6	4700	52	15.0	9.3	780
ISSSTE	15	69	77.2	1035	16	17.3	9.3	277
SEDENA	10	28	46.5	280	10	9.6	8.6	96
Private hospital	55	392^c^	692.6	90,860	55	16.8	9.6	924
Total	200	514		102,802	203	15.4		3099

IMSS *Instituto Mexicano del Seguro Social*; SSA *Secretaría de Salud*; ISSSTE *Instituto de Seguridad y Servicios Sociales de los Trabajadores del Estado*; SEDENA *Secretaría de Defensa Nacional*;

SD standard deviation

^a^Kruskal-Wallis H 52.7, df 5, *p* < 0.000001 for difference between groups

^b^Kruskal-Wallis H 8.5, df 5, *p* = 0.13 for difference between groups

^c^This includes the costs of consultation, medicines and transport as reported by respondents, but excludes other costs associated with private hospitalization such as investigations, special procedures, and bed-day charges.

Table 3 shows an extrapolation of the figures for work and school days lost from dengue in patients and caregivers in the last 12 months to the whole population of the three coastal regions. This extrapolation is justified on the basis that the urban: rural balance in each region in the study sample is similar to that for the whole population in each region [11].

**Table 3:** Reported work or school days lost by dengue patients and their caregivers in the previous 12 months in the baseline survey, with extrapolation to the whole population of each region

Item	Numbers in each region			Total
	Acapulco	Costa Grande	Costa Chica	
Sample population	18,997	17,063	18,342	54,402
Population	789,971	413,793	428,501	1,632,265
*Ambulatory dengue cases*				
Cases reported in sample	483	194	77	754
Projected cases in population	19,749	4552	1714	26,015
Mean days lost per case	11.4	8.8	11.6	10.8
Projected days lost in population	225,142	40,058	19,882	285,082
*Hospitalized dengue cases*				
Cases reported in sample	129	47	30	206
Projected cases in population	5530	1241	857	7628
Mean days lost per case	14.8	15.1	17.4	15.4
Projected days lost in population	81,844	18,739	14,912	115,495
*All dengue cases*				
Projected days lost in population	306,986	58,797	34,794	400,577

### Impact of the Camino Verde intervention

The follow-up survey included 10,491 households, 5349 from 45 intervention sites and 5142 from 45 reference sites. Table 4 shows for intervention and control clusters the proportions of households with at least one dengue case, the dengue case rates per 1000 population, the mean work or school days lost due to dengue for patients and caregivers, and the number of days lost per 1000 population. The proportion of households with at least one dengue case in the last 12 months was lower in the intervention clusters; in this Mexican arm of the trial, the difference was significant at the 9% level. The mean number of days of work or school lost by a dengue case or caregiver was not different between intervention and control clusters, but the days lost per 1000 population were less in intervention clusters because of the reduced number of cases.

**Table 4:** Proportion of households with dengue cases, rate of dengue occurrence and mean number of work/school days lost in intervention and control clusters in last 12 months

	Intervention	Control	Significance of difference
Households with dengue cases	6.1% (326/5349)	7.9% (407/5141)	*p* = 0.09^1^
Dengue case rate in trial populations	19.8/1000	27.1/1000	
Mean number of work/school days lost by the dengue patients	9.1 (SD 7.88)	8.4 (SD 7.31)	*p* = 0.22^2^
Days lost by dengue cases /1000 population	180.18	227.64	
Mean number of work/school days lost by caregivers of dengue patients	9.08 (SD 8.13)	8.26 (SD 7.46)	*p* = 0.12^2^
Days lost by caregivers /1000 population	179.78	223.85	

^1^Cluster t-test. Mean difference − 0.018, 95% CIca −0.040 - 0.004, *t* = −1.685,df = 88

^2^Unpaired t-test

*Extrapolation of days saved by the intervention:*

Saving in days lost by sick people per thousand population = 227.64–180.18 = 47.46 /1000

Saving in days lost by caregivers per thousand population = 223.85–179.78 = 44.07 /1000

Total savings in days lost per thousand population = 47.46 + 44.07 = 91.53 /1000 = 0.09153

As shown in Table 4, the Camino Verde intervention resulted in a saving in days lost for dengue patients and caregivers. The saving was 47.46 days per 1000 population for patients and 44.07 days per 1000 population for caregivers – a total of 91.53 days saved per 1000 population. The estimated population for the three coastal regions of Guerrero is 1,632,265: 789,971 in Acapulco, 413,793 in Costa Grande, and 428,501 in Costa Chica [13]. If the intervention were applied to this whole population, it could be expected to save 149,401 days due to dengue illness (1,632,265 × 0.09153).

Additional file 1: Table S1 shows the reported expenditures on different elements of treatment for dengue cases in intervention and control clusters, for ambulatory and hospitalized cases. The costs per case were similar between intervention and control clusters, but because of the reduced number of cases, the overall costs for every element were lower for intervention than control clusters.

## Discussion

Our study confirms that costs of dengue illness to households are substantial in a dengue endemic area of Mexico, especially when one takes into account work or school days lost. According to the 2012 National Household Income and Expenditure Survey the average monthly income for Mexican households in the lowest income decile was USD185 [14]. Treatment of an ambulatory dengue case by a private physician, for example, would thus consume more than half a month’s income (Table 1) and hospitalization in a private facility would require more than two month’s income from households in this income range, even when not taking into account the full cost to the patient of a private hospital stay (Table 2). These costs are in addition to the loss of 17 workdays which, to lower-income families, can be much more devastating than to those with steadier employment and benefits packages.

In a separate analysis of expenditures on insecticide anti-mosquito products reported in our 2012 impact survey, we found a monthly expenditure of USD6.00 in intervention communities and USD6.83 in reference communities, which represents 3.3% and 3.8% respectively of monthly income for the poorest 10% of the population in 2012 [15].

In 2009 Suaya and colleagues examined the costs of dengue illness in eight countries of Asia and the Americas. Days lost from school or work for non-hospitalised dengue patients and those who took care of them are similar in our study to those reported by Suaya in Guatemala, Venezuela and Malaysia and those reported by the same authors in Guatemala and Malaysia are similar to ours, for patients who went to private hospitals [7].

Undurraga et al. reported higher costs than ours, for hospitalised and non-hospitalised dengue patients [8]. The difference might be because they took into account costs to the government for the health services they provide, and monetized indirect costs, whereas we did not include government services costs and did not monetize indirect costs. Also, our costs for hospitalized cases are under-estimates because they do not include costs beyond consultation, medicines and transport. This is particularly relevant for private hospital stays, for which the total costs to the patients will be much higher than USD392.

The costs of dengue reported by Castro and colleagues from Colombia for both hospitalised and non-hospitalised dengue cases were lower than we found in Mexico [9]. The difference could be because 96% of Colombians in 2011 were covered by some form of health insurance, whereas in our study in Mexico, 28% of the hospitalized patients were treated in private health facilities, and were unlikely to have any form of insurance to cover medical costs.

While household behaviour may be most directly impacted by an understanding of their own immediate costs from cases of dengue fever, citizens should also be aware of the illness’s full societal costs since their taxes are contributing to what government health institutions do to treat dengue cases. Zubieta-Zavala et al. estimated that in 2012 in Mexico costs for patients in the SSA system were USD32.60 in the outpatient setting, and USD490.93 in the hospital setting. For patients enrolled in the IMSS, costs were USD92.03 in the outpatient setting, and USD1644.69 in the hospital setting [16]. They further estimated that the full costs of ideal treatment, according to a recommended treatment protocol, would be importantly higher.

Dengue is just one illness that can befall a household in any given year. Many researchers have examined the burden to households of costs associated with illness in general and the care received for it [17]. In 2006 McIntyre and colleagues conducted a critical review of studies carried out in low- and middle-income countries focusing on the economic consequences for households of illness and health care use. They found that the total economic cost of illness for households was frequently above 10% of household income, a cost that is potentially catastrophic [18]. Our finding on the economic impact of dengue illness on the lowest income families in one area of Guerrero state should be understood as part of a much broader problem.

The study protocol for the Camino Verde [19] trial anticipated a reduction in dengue cases as a primary outcome and savings attributable to prevention of dengue cases as one of its corollaries. The present analysis confirms that this did indeed happen, at least in the Mexico arm of the trial. This was not due to reduced costs per case, but rather to a reduced number of cases. The reduced number of cases (and reduced proportion of households with at least one dengue case) was significant only at the 9% level in our analysis of the Mexican arm of the trial (see Table 4); in the overall trial, with a larger number of clusters, this difference was significant at the 5% level [10]. Had the intervention been applied to the whole population of the three coastal regions, as many as 149,401 work or school days could have been saved. The analysis also confirms monetary savings associated with the Camino Verde intervention, specifically a reduction in the costs of all elements of dengue treatment (Additional file 1).

These findings about household costs and the potential reduction of these costs from the Camino Verde intervention are an important substrate for community discussions and can encourage residents to get involved in the interventions, which may include such things as keeping their water storage containers clean and covered and removing materials from around the household that can allow water to accumulate. Information concerning the costs of dengue illness and the benefits of an alternative approach to the chemical solution were an important part of the “socialisation of evidence for community action” (SEPA) at the heart of the Camino Verde intervention [20, 21].

### Limitations

Dengue cases in the households may have been under-reported. While household respondents probably recalled diagnosed dengue cases that occurred in the last 12 months, some cases might not have been recognised as dengue, especially if they were mild. However, we have no reason to believe that under-reporting would be different between intervention and control sites; the intervention did not include anything about recognition of clinical dengue illness.

As with all self-reported costs, there may be inaccuracies in the costs reported in this study, especially as they were reported by one respondent in the household and could have been incurred up to one year ago. Our question about where individuals received treatment for dengue did not take into account the possibility of their having been treated in more than one place. The fact that the numbers of work or school days lost and the monetary cost *per dengue case* were similar between intervention and control communities does not suggest any important bias in cost reporting related to the intervention.

We did not ask a separate question to those households reporting a member who was hospitalised for dengue in a private facility, inquiring how much they recalled paying in addition to the amount for consultations, medicines and transport. The additional cost is likely to be substantial among the 55 (27.5%) of dengue hospital stays that were in private hospitals. However, it is likely that most of the 55 were from higher than average income families; just three were from households where the household head was not in paid employment and would probably not have had access to IMMS, ISSTE or SEDENA. Unfortunately, we were unable to find any up-to-date estimates of costs for treatment in private hospitals in Mexico, but in countries where comparisons were possible costs of treatment in private hospitals were found to be higher than those in public facilities [7].

## Conclusion

Dengue cases pose an important economic burden on households in a dengue endemic area. The community-based intervention of the Camino Verde trial, because it reduced the number of dengue cases, resulted in a significant reduction in work and school days lost by patients and their caregivers and savings in all aspects of costs of treatment of cases. Applied to the whole population of the coastal regions of Guerrero, the Camino Verde intervention could have saved them over 400,000 work or school days annually.

## Additional file

Additional file 1: Table S1. Estimates of direct and indirect costs of dengue illness cases in the previous year, in intervention and control sites, reported in the follow up survey. (PDF 62 kb)

## Abbreviations

IMSS: Instituto Mexicano del Seguro Social
ISSSTE: Instituto de Seguridad y Servicios Sociales de los Trabajadores del Estado
SEDENA: Secretaría de Defensa Nacional
SSA: Secretaría de Salud
