# Supplementary material for: Household costs of dengue illness: secondary outcomes from a randomised controlled trial of dengue prevention in Guerrero state, Mexico
Source: BMC Public Health. 2017 May 30;17(Suppl 1):411. doi: 10.1186/s12889-017-4304-x (PMC5506602; doi:10.1186/s12889-017-4304-x)
Supplement: Additional file 1: Table S1. — Estimates of direct and indirect costs of dengue illness cases in the previous year, in intervention and control sites, reported in the follow up survey. (PDF 62 kb) [file 12889_2017_4304_MOESM1_ESM.pdf]

Additional File 1. Table S1. Estimates of direct and indirect costs of dengue illness cases in the previous year, in intervention and control sites, reported in the follow up survey

	**Intervention sites (population 22,616)**				**Control sites (population 21,410)**			
**A. Ambulatory patients**								
1. Direct Costs								
	Mean cost per case	Total No. cases	No. cases / 1000	Cost /1000	Mean cost per case	Total No. cases	No. cases / 1000	Cost /1000
Consultation	10.6	151	6.68	**70.8**	15.6	197	9.20	**143.5**
Medicines	28.5	219	9.68	**276.0**	26.5	292	13.63	**361.4**
Transport	7.3	220	9.73	**71.0**	8.5	268	12.52	**106.4**
2. Indirect Costs								
	Mean days per case	Total No. cases	No. cases / 1000	Days lost /1000	Mean days per case	Total No. cases	No. cases / 1000	Days lost /1000
School or work days lost by the patient	8.5	377	16.67	**141.7**	7.7	458	21.39	**164.7**
School or work days lost by the caregiver(s)	8.2	310	13.71	**112.4**	7.7	372	17.38	**133.8**
**B. Hospitalized patients**								
1. Direct costs								
	Mean cost per case	Total No. cases	No. cases / 1000	Cost /1000	Mean cost per case	Total No. cases	No. cases / 1000	Cost /1000
Consultation	41	16	0.70	**28.7**	32	22	1.02	**32.64**
Medicines	98	19	0.84	**82.3**	78	44	2.05	**159.90**
Transport	19.3	46	2.03	**39.2**	19	67	3.12	**59.28**
2. Indirect costs								
	Mean days per case	Total No. cases	No. cases / 1000	Days lost /1000	Mean days per case	Total No. cases	No. cases / 1000	Days lost /1000
School or work days lost by the patient	14	44	1.94	**27.16**	12	69	3.22	**38.64**
School or work days lost by the caregiver(s)	15	43	1.90	**28.50**	10	78	3.64	**36.40**
